# Lysophosphatidyl Choline Induced Demyelination in Rat Probed by Relaxation along a Fictitious Field in High Rank Rotating Frame

**DOI:** 10.3389/fnins.2017.00433

**Published:** 2017-08-03

**Authors:** Lauri J. Lehto, Aloma A. Albors, Alejandra Sierra, Laura Tolppanen, Lynn E. Eberly, Silvia Mangia, Antti Nurmi, Shalom Michaeli, Olli Gröhn

**Affiliations:** ^1^Department of Neurobiology, A.I. Virtanen Institute for Molecular Sciences, University of Eastern Finland Kuopio, Finland; ^2^Department of Neurobiology, Center of Magnetic Resonance Research, University of Minnesota Minneapolis, MN, United States; ^3^Charles River Discovery Services Kuopio, Finland; ^4^Division of Biostatistics, University of Minnesota Minneapolis, MN, United States

**Keywords:** RAFF, DTI, relaxation, myelin, demyelination, white matter damage, lysophosphatidyl choline

## Abstract

In this work a new MRI modality entitled Relaxation Along a Fictitious Field in the rotating frame of rank 4 (RAFF4) was evaluated in its ability to detect lower myelin content in lysophosphatidyl choline (LPC)-induced demyelinating lesions. The lesions were induced in two areas of the rat brain with either uniform or complex fiber orientations, i.e., in the corpus callosum (cc) and dorsal tegmental tract (dtg), respectively. RAFF4 showed excellent ability to detect demyelinated lesions and good correlation with myelin content in both brain areas. In comparison, diffusion tensor imaging metrices, fractional anisotropy, mean diffusivity and axonal and radial diffusivity, and magnetization transfer (MT) metrices, longitudinal relaxation during off-resonance irradiation and MT ratio, either failed to detect demyelination in dtg or showed lower correlation with myelin density quantified from gold chloride stained histological sections. Good specifity of RAFF4 to myelin was confirmed by its low correlation with cell density assesed from Nissl stained sections as well as its lack of sensitivity to pH changes in the physiological range as tested in heat denaturated bovine serum albumin phantoms. The excellent ability of RAFF4 to detect myelin content and its insensitivity to fiber orientation distribution, gliosis and pH, together with low specific absorption rate, demonstrates the promise of rotating frame of rank *n* (RAFFn) as a valuable MRI technique for non-invasive imaging of demyelinating lesions.

## Introduction

The quantitative assessment of myelin in the brain is highly important for both diagnostic and monitoring purposes of a variety of disorders, including multiple sclerosis (MS), traumatic brain injury (TBI), and stroke. However, conventional magnetic resonance imaging (MRI) approaches currently used to detect demyelination are suboptimal in their ability to detect myelin. To date, the identification of specific pathological processes involving demyelination by conventional MRI methods is not possible. This is because many different pathological processes result in similarly-appearing lesions in conventional MR, e.g., inflammatory demyelination, infection, stroke or tumor may all appear as bright lesions on T_2_-weighted images.

Advanced MRI modalities such as diffusion tensor imaging (DTI) (Schmithorst et al., 2002), magnetization transfer (MT) (Does et al., 1998), T_1_*/*T_2_ ratio (Glasser and Van Essen, 2011), multiexponential T_2_ (McCreary et al., 2009), and direct detection using ultra-short echo time (Wilhelm et al., 2012) have potential in assessing tissue organization and myelin. However, these methodologies just partially address the problem of myelin detection (Glasser and Van Essen, 2011; Nossin-Manor et al., 2013). For instance, although DTI is sensitive to tissue microstructure, it is not specific to myelin (Le Bihan and Johansen-Berg, 2012), because metrics derived from DTI data are influenced also by the underlying macroscopic level organization of myelinated fibers. On the other hand, MT has contributions from T_1_ in different water pools, and the results are dependent on experimental parameters such as off-resonance frequency and saturating B_1_ field strength. For example, the MT ratio (MTR) showed equally strong correlations with both the degree of myelin loss and the extent of axonal loss in postmortem samples (Schmierer et al., 2004). T_1_/T_2_ ratio has been shown to provide good correlation with myelin in normal gray matter (Glasser and Van Essen, 2011), but just like T_1_ and T_2_, it is influenced by the change of multiple different factors such as edema and paramagnetic ion concentration in the pathological brain (Pirko and Johnson, 2008; Beer et al., 2016). Furthermore, in a recent study by Dula and co-workers (Dula et al., 2010), myelin water fraction, an often cited multiexponential T_2_ relaxation metric, varied by almost a factor of 2 between two regions in the spinal cord while the myelin volume fractions differed by only approximately 12%. The proposed explanation for such observation relied on variation in microanatomy and intercompartmental water exchange. Overall, no currently available MRI technique or other noninvasive methods have proven capable of specifically signaling the myelin loss.

Recently, we introduced a novel MRI relaxation method entitled Relaxation Along a Fictitious Field (RAFF) (Liimatainen et al., 2010, 2011) in the rotating frame of rank *n* (RAFFn) (Liimatainen et al., 2015). With RAFFn, the fictitious fields are created by a non-adiabatic modulation of the RF amplitude and frequency. The particular case of the fictitious fields **H**_n_ of rank n which are stationary in the rotating frame of rank (n-1) was detailed in (Liimatainen et al., 2015). We have shown that frequency modulated pulses operating in the nonadiabatic regime in the 1st rotating frame, and thus producing a large fictitious magnetic field in the 2nd rotating frame, can be used for generating novel MRI contrast in living samples. RAFFn is conceptually different from more conventional rotating frame MRI methods. With continuous wave spin lock and adiabatic T_1ρ_ and T_2ρ_ methods, relaxations are governed solely by longitudinal or transverse relaxations in the rotating frame. On the other hand, relaxations during RAFF2 comprise contributions from both T_1ρ_ and T_2ρ_ relaxation pathways. Similarly, RAFFn comprises both T_1ρ_(n) and T_2ρ_(n) relaxation channels. Moreover, RAFFn is also conceptually different from chemical exchange saturation transfer (CEST) and/or MT since RAFFn is sensitive to all contributing relaxation mechanisms including dipole-dipole interactions, diffusion in local field gradients and exchange between spins with different chemical shifts and the cross-relaxations. On the other hand, CEST and MT provide direct measure of the saturation transfer or exchange between off-resonance spins and water. We have shown that for adiabatic T1ρ the contribution of cross relaxations (origin of MT) between spins within the bandwidth of the adiabatic pulse is minor (Michaeli et al., 2008). The contribution of off-resonance saturation to RAFFn relaxation may originate also from side bands at frequencies defined by the duration of the RAFFn pulses (Liimatainen et al., 2015), a mechanism which may enhance sensitivity of RAFFn to multiple spin pools within the myelin component. These frequencies are determined by the assembling of the RAFFn pulses into P-packets, which refocuses the magnetization in the form of rotary echo (Solomon, 1959). Formation of the rotary echoes in the rotating frame during RAFFn pulses is an additional distinction from MT and CEST techniques.

RAFFn generates flexible MRI contrast with low specific absorption rate (SAR) and is thus particularly safe for human applications. We have demonstrated that by changing the rank of the rotating frame and the orientation of the fictitious field in the rotating frame of reference, RAFFn MRI contrast can be sensitized to various motional regimes. In particular, RAFF4 and RAFF5 are exquisitely sensitive to slow/ultra-slow motion characterized by the correlation times (τ_c_) in the millisecond time scale. This is important for sensitive and specific myelin detection as the highly organized structure of myelin contains multi-compartment water environments: myelinic, intra-axonal, and interaxonal. These pools are characterized by different molecular mobilities and T_2_ relaxation components which correspond to ultra-short (50 μs–1 ms), short- (1–50 ms), intermediate (50 ms–0.5 s), and long-lived T_2_ components (>0.5 s) (Does et al., 1998; Bonilla and Snyder, 2007). The short T_2_ component is attributed mainly to myelin water, whereas an ultra-short component is thought to arise from carbon-bound methylene protons. Extensive experimental and theoretical studies by Mefed and coworkers have demonstrated that relaxation measurements in high–rank rotating frames of T_1ρρ_ and T_1ρρρ_ (second and third frame longitudinal relaxations, respectively) allow the probing of slow-ultra slow motion with the characteristic correlation times up to 10^−1^–10^−3^ s (Mefed, 1999, 2001). Our previous analysis, in agreement with those by Mefed, also shows that the sensitivity of RAFF4 and RAFF5 is shifted to the motional regime in the millisecond time scale as compared to adiabatic T_1ρ_ (Michaeli et al., 2006) which has a maximal sensitivity to the correlation times in the microsecond time scale (Satzer et al., 2015).

Utilizing the sensitivity of RAFF4 and 5 to slow molecular motion, we have recently embarked on measuring myelin content in the normal brain and in complex mucopolysaccharidosis type I (MPS-I) pathology including demyelination (Satzer et al., 2015; Hakkarainen et al., 2016). Importantly, the highest correlation between relaxation time constants and myelin content as assessed by quantitative histology was achieved with RAFF4 and RAFF5 techniques, as compared to other relaxation based contrasts (Satzer et al., 2015; Hakkarainen et al., 2016).

In the present work, we further focused on intracranial lysophosphatidyl choline (LPC) injections in the rat brain that have been used previously to model focal demyelination lesions in MS (Waxman et al., 1979; Ransohoff, 2012). LPC injection causes demyelination in the white matter within 3-5 days with only mild inflammatory response, thus allowing a clean model to study demyelination. Another important feature of the model is that location of the white matter lesion can be freely chosen, thus allowing to study the influence of underlying macroscopic structure of the myelinated fibers on MRI contrasts. The optimal MRI contrast for detection of demyelination would be sensitive to the amount of myelin regardless of macroscopic level anisotropy.

The aim of this study was to characterize the ability of RAFF4 to detect demyelination processes in presence of different underlying tissue macrostructure and to compare that with some of the approaches currently used to detect white matter lesions in advanced human studies. To achieve these goals, we induced demyelination by injecting LPC in the rat corpus callosum (cc) and dorsal tegmental tract (dtg), measured RAFF4, DTI and MT parameters, and performed histology validation to assess the ability of the various MRI modalities to detect demyelination. Furthermore, influence of pH on RAFF4 contrast was evaluated using bovine serum albumin containing phantoms.

## Materials and methods

### Animal model

Male Spraque Dawley rats (*n* = 21, Charles River, Germany; 300–350 g) were used in the experiments. Rats were group housed with a preserved 12 h light/12 h dark cycle and *ad libitum* access to food and water. All animal procedures were approved by the Animal Ethics Committee of the Provincial Government of Southern Finland, and conducted in accordance with the guidelines set by the European Commission Directive 2010/63/EEC.

All surgical procedures were done under inhalation anesthesia using 1.8–2.2% isoflurane in 30%/70% O_2_/N_2_O. To induce demyelinated lesions, stereotaxic injections of the LPC solution (volume of 1.5 μl; concentration: 10 mg/ml; L-α-lysophosphatidylcholine from egg yolk; L-4129 Sigma-Aldrich, St. Louis, USA) were performed in selected areas of the rat brain, chosen based on their myeloarchitecture. The injections were placed either in the cc (*n* = 6 and *n* = 4, LPC and vehicle, respectively) at the stereotaxic coordinates: 0.4 mm posterior from bregma, 1.4 mm lateral from the midline, and 2.6 mm from the skull. For the dtg (*n* = 6 and *n* = 4, LPC and vehicle, respectively) the injection coordinates were at 6.3 mm posterior from bregma, 0.8 mm lateral from the midline, and 4.3 mm from the skull. The cc represents a major white matter bundle with the parallel organization of myelin bundles, while dtg is a typical example of more complex myeloarchitecture with a high density of heterogeneously oriented axons. Control animals underwent identical protocol but were injected with 1.5 μl of vehicle solution [0.1 M sodium phosphate buffer (PBS)] instead of LPC.

### MRI

All the animals were imaged 3 days after injection, when there is already significant demyelination in this animal model, while inflammatory reaction and remyelination typically develops later (Waxman et al., 1979). All MR experiments were performed using a horizontal 7 T magnet system (Bruker Pharmascan, Entlingen, Germany).

The sites of injections were localized using T_2_-weighted fast spin-echo (FSE) images with the following parameters: TR = 4 s, echo spacing 12 ms, TE_eff_ = 48.0 ms, n_echo_ = 8, FOV = 25.6 × 25.6 mm^2^, matrix size = 256 × 256, number of slices = 20 and slice thickness = 0.75 mm. The imaging slice for RAFFn, MT and DTI (middle slice) was centered to the FSE slice next to the injection site to avoid including mechanical damage induced by the injection.

### RAFFn technique

A detailed description of the RAFFn technique was presented elsewhere (Liimatainen et al., 2015). We chose to use RAFF4, as RAFF4 and 5 showed highest correlation to myelin content in normal rat brain in our previous study (Hakkarainen et al., 2016), and as RAFF4 relaxation is faster than RAFF5 thus requiring shorter pulse train duration than RAFF5. With RAFFn, the *sine*/*cosine* pulses were used for the modulation of the amplitude and frequency, respectively. The orientation of the effective field of RAFFn in the rotating frame of rank (n-1) relative to the *Z*^(n−1)^ axis of quantization is defined by the angle α^(*n*)^, as was previously detailed (Liimatainen et al., 2010, 2011, 2015). Here, the α^(*n*)^ was set to 45° in each rotating frame of rank n-1, and the amplitude of the effective field H_n_ was maintained at the same level. During RAFFn pulses, the rotary echoes are generated when using four pulse elements assembled into a *P*-packet according to the scheme *PP*^−1^*P*_π_$P_{\pi }^{-1}$ (Liimatainen et al., 2010). An instantaneous flip of the effective field H_n_ is indeed performed during each *P*-packet to ensure refocusing of **M** on the *Z*^(n−1)^ axis and to form rotating frame rotary spin echo (Solomon, 1959). The peak RF amplitude of RAFF4 pulses was γ*B*_1_ = 323 Hz. The time duration of each *PP*^−1^*P*_π_$P_{\pi }^{-1}$ packet defined as $T_{\text{p}}=4\pi /\left(\sqrt{2}\omega_{1}^{\max}\right)$ was set to 4.525 ms. The signal intensity decay was measured by incremental pulse trains of *P*-packet, with an inversion pulse to account for steady state. RAFF4 pulse train durations were 0, 27, 54, 81, and 108 ms.

Fast spin-echo (FSE) was used as a readout pulse sequence with TR = 4 s, TE_eff_ = 8.3 ms, n_echo_ = 8, FOV = 32.0 × 32.0 mm^2^, matrix size = 256 × 256, number of slices = 1 and slice thickness = 0.5 mm, leading to total acquisition time 21 min 20 s for one relaxation time constant map.

### Comparator techniques: DTI and MT

MT measurements were conducted using the same FSE readout sequence as described above for RAFF4. The modified inversion MT protocol with two consecutive acquisitions was used (Mangia et al., 2011). First, the signal decay during off-resonance irradiation with **M** oriented along +Z was acquired, and, second, the signal recovery was measured when **M** was inverted along −Z. A square saturation pulse with γ*B*_1_ = 200 Hz was placed 8 kHz off-resonance with an incrementing pulse duration of 0, 0.3, 0.6, 0.9, and 1.2 s. Total acquisition time was 21 min 20 s. T_1sat_, steady state magnetization M_SS_ and magnetization in fully relaxed state, M_0_, were solved using pixel-by-pixel analysis with monoexponential decay and rise functions to the same steady state value, as described in Mangia et al. (2011). MTR was calculated as MTR = 1–M_SS_/M_0_.

For DTI, segmented spin-echo EPI was used with TR = 2 s, TE = 30.0 ms, n_shots_ = 6, number of averages = 12, FOV = 26.5 × 18.0 mm^2^, matrix size = 212 × 144, number of slices = 9, slice thickness = 0.5 mm, b = 1,000 s/mm^2^, diffusion directions = 42 leading to total acquisition time of 1 h 55 min. Mean diffusivity (MD), fractional anisotropy (FA), radial and axial diffusivity (RD, AD) maps were calculated from DTI data.

### ROI analysis with MRI

Relaxation time constant maps and parametric maps from MT and DTI data were calculated in MATLAB (MathWorks, Natick, MA). The regions of interest (ROIs) were hand-drawn using the Aedes software package (http://aedes.uef.fi). Six ROIs were drawn in the cc, three contralateral (1, 2, and 3) and three ipsilateral (4, 5, and 6) to the injection site (**Figure 3**). Mean values of ROI 4 further averaged over LPC or vehicle animals were used to evaluate the relative contrast (RC) of each of the different MRI metrics between LPC and vehicle injected animals. For metrics with lower intensity in vehicles, RC = [LPC–vehicle]/LPC ^*^ 100%. For metrics with higher intensity in vehicles, RC = [vehicle–LPC]/vehicle ^*^ 100%. This approach ensures that RC is comparable between techniques regardless of the direction of change between LPC and vehicle animals. Further, by dividing the difference with the higher value makes RC a conservative approach as it has a maximum of 100%. In addition, relative contrast-to-noise ratio (RCNR) was calculated so that RCNR = [higher value–lower value]/SD(mean values of ROI4 of the vehicles). To have a broader dynamic range of myelinated tissue, all 6 ROIs were used to calculate correlations between MRI and myelin content based on histological stainings as described below. For the dtg, an ROI covering the lesion was drawn and copied to the contralateral side to the corresponding anatomical location. For the vehicle injected animals, a 3-by-3 voxel ROI was drawn at the injection site and copied to the same anatomical location on the contralateral side. RC between LPC and vehicle injected animals were then calculated as for the cc, using the contralateral ROIs.

### Histological procedures

Immediately after the MRI scans, all the animals were transcardially perfused with 0.9% NaCl (30 ml/min, 2 min, 4°C), followed by 4% paraformaldehyde (PFA) solution in 0.1 M phosphate buffer (pH 7.4, 30 ml/min, 25 min, 4°C). Fixed brains were removed from the skull, and post-fixed for 4 h in 4% PFA. Then, the brains were cryoprotected in 20% glycerol in 0.02 M potassium phosphate-buffered saline (pH 7.4) for 36 h, and frozen in dry ice. The frozen brains were stored at −70°C until sectioning.

The brains were cut in five series of 30-μm thick coronal sections using a sliding microtome. The first series of sections was stored in 10% formalin, at room temperature. The series from second to fifth were stored in a cryoprotectant tissue-collecting solution (30% ethylene glycol, 25% glycerol in 0.05 M PBS) at −20°C until further processing.

The first series of sections was stained with Nissl (thionin) to study cytoarchitecture, cell death and gliosis. The second series of sections was stained with gold chloride to assess the myeloarchitecture. For myelin staining, sections were mounted on gelatin-coated slides and dried at 37°C. They were then incubated in a 0.2% gold chloride solution (HAuCl_4_•3H_2_O, G-4022 Sigma-Aldrich, St. Louis, USA) in 0.02 M PBS (pH 7.4) containing 0.09% NaCl for 7–8 h at room temperature in the dark. Then, the slides were washed twice for 4 min in 0.02 M PBS in 0.09% NaCl and placed in a 2.5% sodium thiosulfate solution for 5 min. After three 10 min washes in the buffer solution, sections were dehydrated through an ascending series of ethanol, cleared in xylene and coverslipped with DePeX (BDH, Laboratory Supplies, Dorset, UK).

### Histological analyses

High-resolution photomicrographs of both myelin- and Nissl-stained sections of the cc and the dtg were obtained using a light microscope (Leica DMRB, Wetzlar, Germany) equipped with a digital camera (DXM1200F, Nikon Instruments Inc., Japan). Three consecutive sections of these areas were analyzed covering a volume of 450 μm that corresponded to the slice thickness in MRI.

The optical density on myelin- and Nissl-stained sections was quantified using ImageJ software (version 1.41 http://rsb.info.nih.gov/ij/). For cc and dtg, ROIs were drawn corresponding in location, size and shape to the MRI ROIs. Optical density (OD) was averaged for each ROI over three consecutive sections to cover the volume analyzed in MRI. After conversion of the images to gray-scale, OD values were obtained from the ROIs. For correction of possible staining differences between sections and brains, OD values from healthy reference areas were obtained. For the cc lesions, white matter in the striatum was used as a reference and for dtg lesions, cc (in the sections of dtg) was used as a reference.

### Transmission electron microscopy (TEM)

One extra rat, injected with LPC as described above, was used to study the ultrastructure of the myelin sheaths in the corpus callosum 3 days after the LPC injection using TEM. T_2_-weighted images were acquired to verify the presence and consistency of the demyelinated lesion. After MRI, the rat was perfused using 0.9% NaCl (30 ml/min) for 2 min followed by 4% PFA (30 ml/min) in 4°C for 50 min. The brain was removed from the skull, post-fixed in 4% PFA/1% glutaraldehyde overnight at 4°C, and sectioned in 1-mm thick coronal sections in a brain matrix. One 1-mm thick section matching the level of the MRI analysis was selected based on T_2_-weighted images. The corpus callosum of this section was dissected into eight small portions from the level of the injection site to the same level on the healthy contralateral side. These corpus callosum samples were incubated with 4% PFA in 0.1 M cacodylate buffer (pH 7.4) at 4°C for an overnight. Then, the samples were rinsed in 0.1 M cacodylate buffer 5 min three times, followed by a post-fixation in 1% osmium tetroxide (OsO_4_) in 0.1 M cacodylate buffer for 2 h. After that, the samples were again rinsed in 0.1 M cacodylate buffer for 5 min three times. Then, the samples were dehydrated in ascending percentage of alcohols for 10 min, and the last incubation was propylene oxide twice for 10 min. The samples were then infiltrated with a 1:1 mixture of propylene oxide and LX-112 resin (Ladd Research Industries Inc., USA) for 1 h, followed by incubation in LX-112 resin overnight. The embedding was done with fresh LX-112 resin in molds. The polymerization of the samples was done in an oven at 37°C for 24 h, and then at 60°C for 48 h.

Once the samples were embedded in the resin, four semi-thin sections of 1 μm were sectioned and stained with Toluidine blue. Toluidine blue stained sections were used to study the cyto- and myeloarchitecture of the samples, and to guide TEM imaging. Six ultrathin sections were cut for TEM and mounted in three different grips. Imaging was done in a transmission electron microscope JEOL JEM-2100F using magnifications between 250 and 10,000x. Photomicrographs were taken using a digital camera connected to the microscope.

### Bovine serum albumin phantoms

Heat denaturated bovine serum albumin samples (BSA) were used to investigate influence of pH on RAFF4. Heat denaturated BSA has been shown to provide a good model to study influence of pH modulated proton exchanges as relaxation mechanism in tissue, while glutaraldehyde crosslinking blocks a significant portion of exchangeable NH-groups in the protein (Mäkelä et al., 2001). BSA (fraction V, Sigma Chemicals, St. Louis, MO) was dissolved in 0.1 M Tris (pH 7.4) to yield a 10% solution. The pH was adjusted to be 6.2, 6.6, 7.0, 7.4, and 7.8 in five BSA samples, respectively, using HCl or NaOH. BSA phantoms were exposed to 65°C for 3 min for heat denaturation.

The pH phantoms were imaged using a horizontal 9.4 T magnet (Magnex Scientific Ltd., Abington, UK) interfaced to a Varian (Agilent) DirectDrive console (Agilent Technologies, Santa Clara, CA). RF transmission and signal reception were carried out using a single loop coil with 20 mm diameter. T_2_-weighted FSE MR imaging was used as a readout with the parameters as follows: TR = 4 s, TE_eff_ = 55 ms, matrix size 256 × 256, FOV = 25.6 × 25.6 mm^2^, 8 echoes with 8 ms echo spacing, initial TE = 10 ms, one slice with slice thickness 1 mm. The settings of RAFF4 were same as for *in vivo* studies described above. T_2_-maps were measured using double spin-echo with two 3 ms adiabatic full passage pulses and echo times of 5, 7, 15, 23, 31, 39, and 63 ms.

### Statistical analyses

The Mann-Whitney *U*-test was used to compare LPC injected animals to vehicles. The contribution of myelinated axons and cell density to the MRI metrics was assessed using Pearson's linear correlation (two-tailed) between pooled ROI analysis results from MRI and histology of myelin and Nissl stained sections, respectively. ROIs from both LPC and vehicle injected animals were included in the analysis including contralateral and ipsilateral ROIs. Correlation was calculated separately for all data, cc data and dtg data. False discovery rate (FDR) correction was done to adjust *p*-values for inflation of error due to multiple testing.

## Results

### Corpus callosum–demyelination in a major white matter track with parallel axons

#### MRI

**Figure 1:**
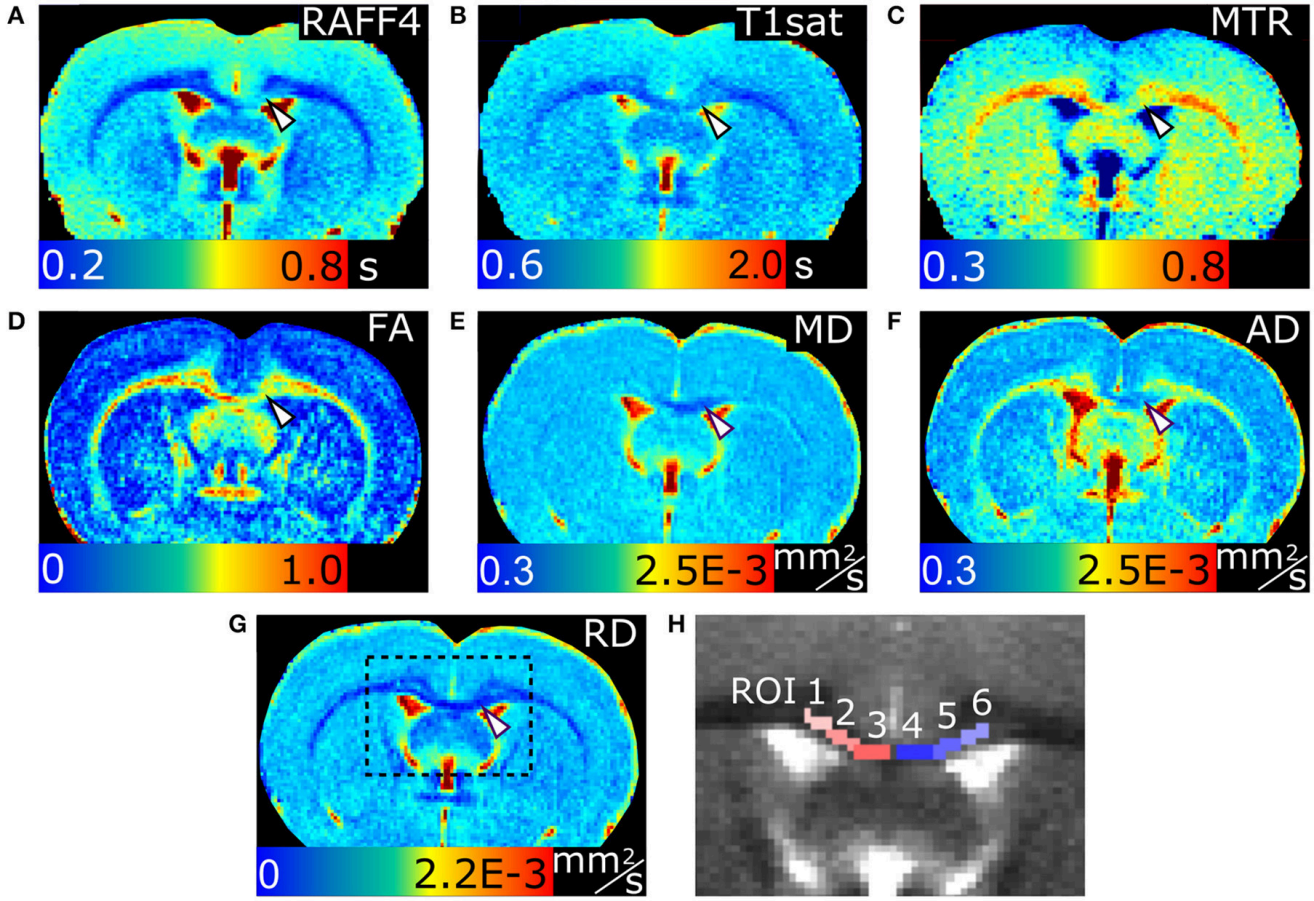
MRI parameter maps of a representative rat, 3 days after an LPC injection into the corpus callosum. Relaxation time constant map of RAFF4 **(A)**, T_1sat_ **(B)**, magnetization transfer ratio, MTR **(C)**, fractional anisotropy, FA **(D)**, mean diffusivity, MD **(E)**, and axial and radial diffusivity, AD and RD **(F,G)**. Representative examples of ROIs for analyzing lesions in the corpus callosum are shown on a grayscale RAFF4 map in **(H)**. White arrowhead points to demyelinated lesion in **(A–G)**.

Representative parametric maps from relaxation, MT and diffusion data obtained from a rat with LPC injection in the cc are shown in Figure 1. The imaging slice was chosen to be 0.75 mm posterior from the injection site to avoid the influence of mechanical damage caused by the injection needle. The lesion was clearly visible in all quantitative MRI maps as well as in myelin and Nissl stained histological slices on the ipsilateral site close to the injection site. In all animals, the lesion was located within the cc, extending ipsilaterally 1.95 ± 0.24 mm (mean ± SD; based on T_2_-weighted images) from the midline and contralaterally 0.05 ± 0.15 mm from the midline in mediolateral direction, due to diffusion of the LPC along the tract.

**Figure 2:**
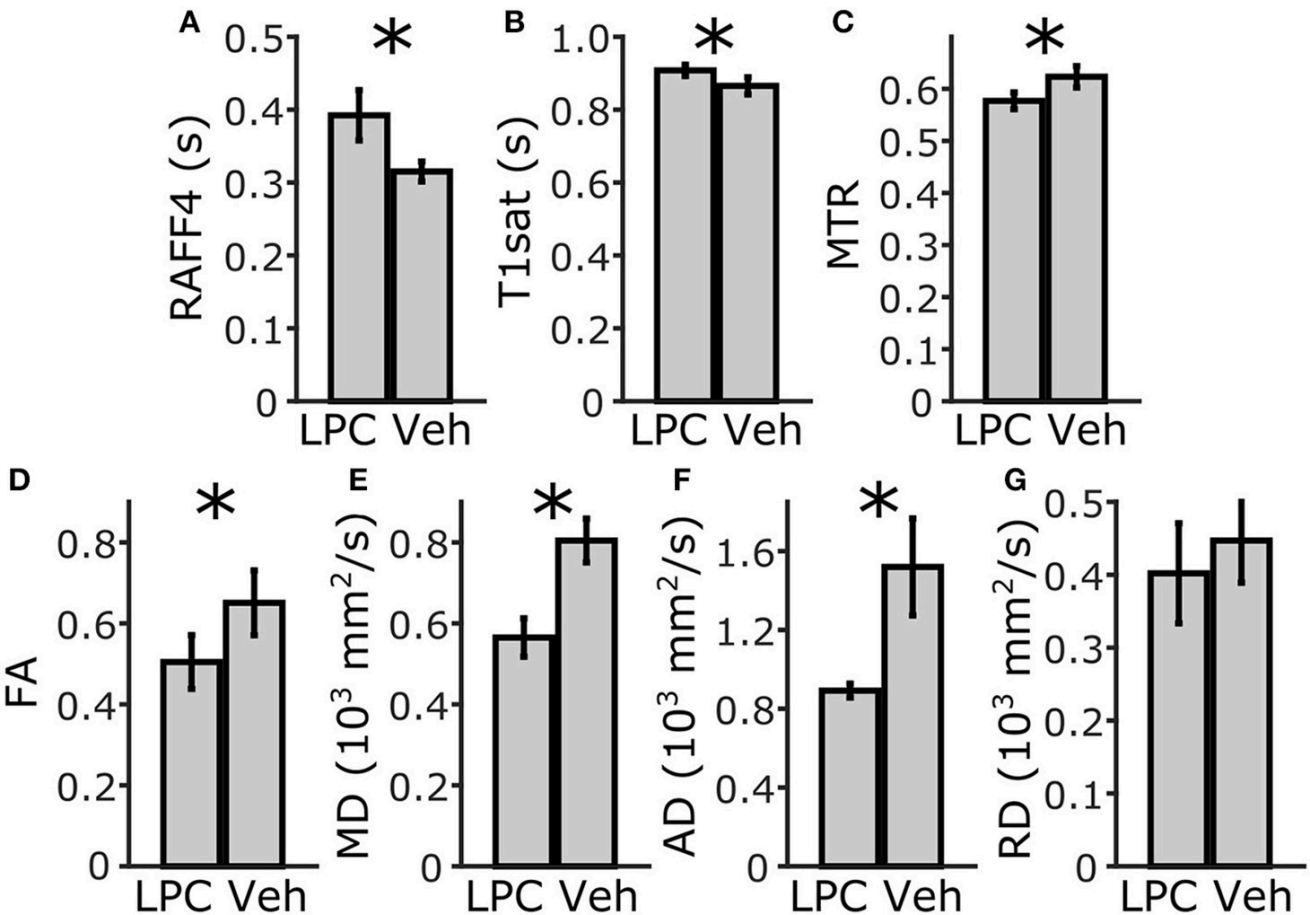
ROI analysis of MRI parameters in the corpus callosum, ipsilateral side. Mean and SD using RAFF4 **(A)**, T_1sat_ **(B)**, magnetization transfer ratio, MTR **(C)**, fractional anisotropy, FA **(D)**, mean diffusivity MD **(E)**, and axial and radial diffusivity, AD and RD **(F,G)** values obtained from LPC-induced lesion ROI 4 in the corpus callosum of LPC injected (*n* = 6) rats and from the corresponding ROI in the vehicle injected (*n* = 4) rats. Statistical significance; ^*^ < 0.05 (FDR adj. for testing 7 MRI parameters), Mann-Whitney *U*-Test.

**Figure 3:**
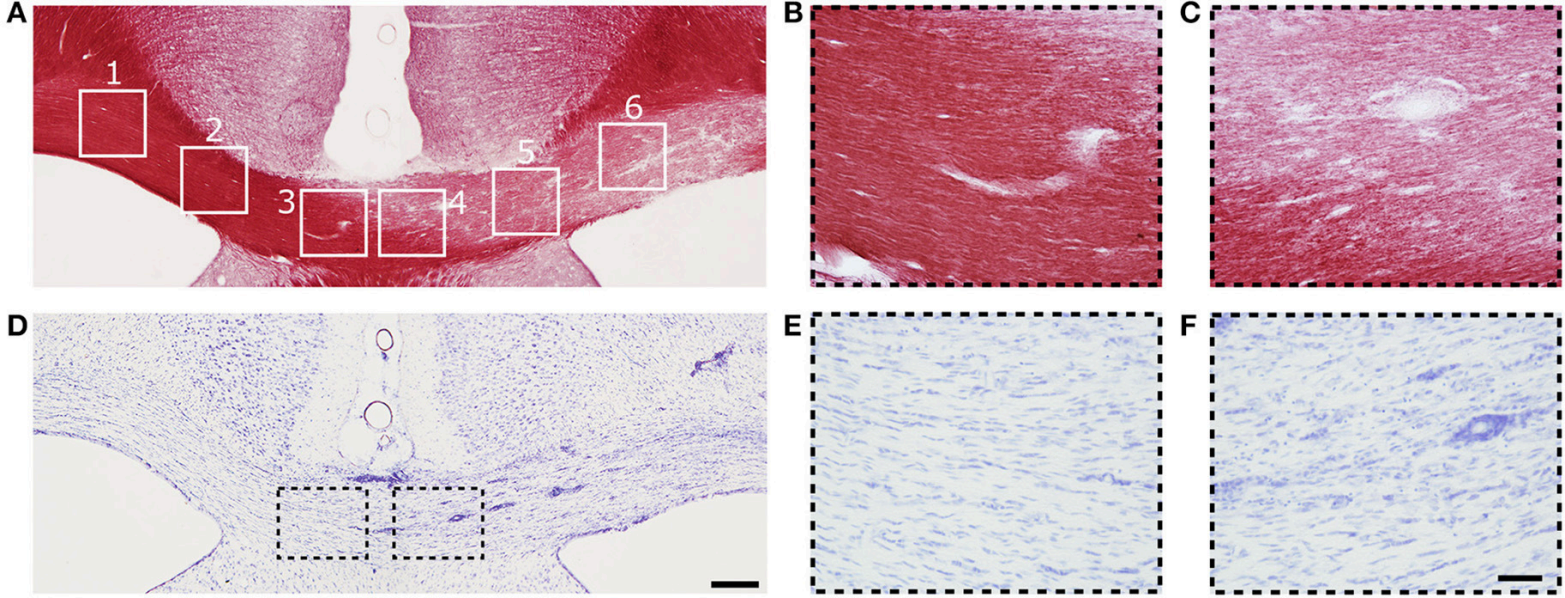
**(A)** Representative photomicrograph of myelin-stained sections of the corpus callosum. **(B,C)** are high magnification images of ROIs 3 and 4 from **(A)**. **(D)** Representative photomicrographs of Nissl-stained sections of the corpus callosum. **(E,F)** Are high magnification images of ROIs 3 and 4 in **(D)**. Scale bars: **(D,F)** 200 and 50 μm, respectively.

In the ROI analysis, all MRI metrics showed statistically significant differences (*p* < 0.05) between lesions induced by LPC in the ipsilateral cc (ROI 4, in **Figure 3**) and the corresponding area in vehicle animals (Figure 2). The RC observed with RAFF4 was 19.7%. The RC of MTR and T_1sat_ were 7.4 and 4.7%, respectively, thus exhibiting smaller RC than RAFF4. On the other hand, MD and FA showed the largest RC: 29.8 and 22.5%, respectively. Reduced MD and FA were explained by decrease in axial diffusivity (AD) by 41.3%, while radial diffusivity (RD) did not show a statistically significant difference, consistent with axonal damage and contradicting the typical pattern of diffusion changes often associated with demyelination (Song et al., 2005). While RC of DTI metrics were higher than that of RAFF4, RCNR of RAFF4 was higher at 5.7 in comparison to 4.4, 1.8, and 2.6 of MD, FA and AD, respectively. Correspondingly, RCNR for MTR and T_1sat_ was 2.3 and 1.8 respectively.

#### Histology and electron microscopy

An example of myelin- and Nissl-stained section from the same animal as was presented for MRI in Figure 1 is shown in Figure 3. Three days after LPC injection in the cc, a demyelinated area over the whole ipsilateral cc was observed (ROI 4 to 6) while the myelin content on the contralateral side appeared normal (ROI 1 to 3, Figure 3A). Also ROI 4, closest to the midline in the ipsilateral side still presented signs of demyelination (Figure 3C), which gradually decreased toward the contralateral side and ROI 3 (Figure 3B).

On Nissl-stained sections, a slightly increased cell density was observed on the demyelinated area (Figure 3D), which can be attributed to mild gliosis. The cell density was higher in ROI 6 and decreased toward the midline (ROI 4) and the contralateral side (ROI 3) (Figure 3D). Mild gliosis overlapped with the demyelinated area observed in myelin staining (Figure 3A). Gliosis was diffusely distributed; however, a high number of cells were found surrounding the blood vessels (Figure 3F). On the contralateral side to the injection site, the density and distribution of cells appeared normal (Figure 3E).

**Figure 4:**
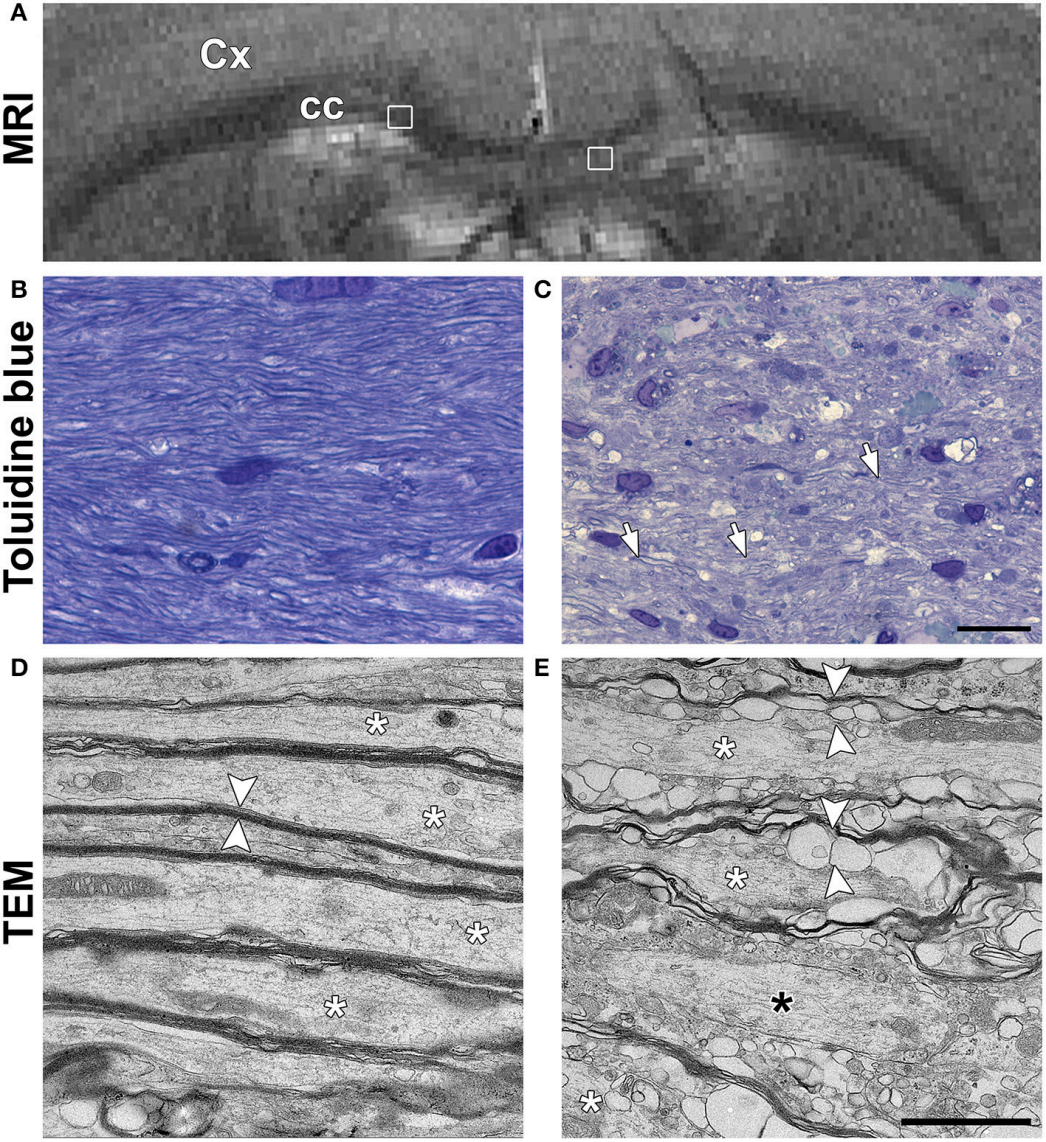
**(A)** *In vivo* T_2_-weighted image of a rat used for the ultrastructural analysis using transmission electron microscopy (TEM). White squares indicate the location of the images shown in **(B,C)**. Representative photomicrographs of Toluidine blue stained sections of the normal corpus callosum **(B)** and demyelinating lesion **(C)**. White arrows in panel C point at axons with thin myelin sheath. Scale bar in **(B,C)** is 20 μm. Representative photomicrographs obtained in TEM are shown for the normal corpus callosum **(D)** and demyelinated lesion **(E)**. White asterisks indicate individual axons in both **(D,E)**. In **(D)**, axons have normal and packed myelin sheaths (white arrowheads). In **(E)**, the myelin sheaths appear loosely packed and full of pockets (white arrowheads). A black asterisk indicates an axon without myelin sheath. Scale bar in **(D,E)** is 200 nm.

In order to understand the unexpected diffusion results in cc, samples from one additional animal underwent TEM. T_2_-weighted imaging confirmed that the location and extent of the LPC lesion was comparable to lesions in other cc injected animals (Figure 4A). Toludine blue staining showed clearly decreased staining intensity around the axons in the ipsilateral cc when compared to the contralateral cc, corroborating demyelination (Figures 4B,C). In TEM, axonal myelin sheaths appeared thick and tightly packed on the contralateral cc. Intra-axonal space appeared normal with only a few membranes or organelles, such as mitochondria, that could restrict diffusion along axons (Figure 4D). In the demyelinating ipsilateral cc, axons were either completely demyelinated or surrounded by thin and disorganized myelin sheaths (Figure 4E). In pockets between the myelin sheaths, vacuoles and myelin debris were observed around all the demyelinated axons, forming more boundaries along the axonal length.

### Dorsal tegmental tract–demyelination in an area with a complex myeloarchitecture

#### MRI

**Figure 5:**
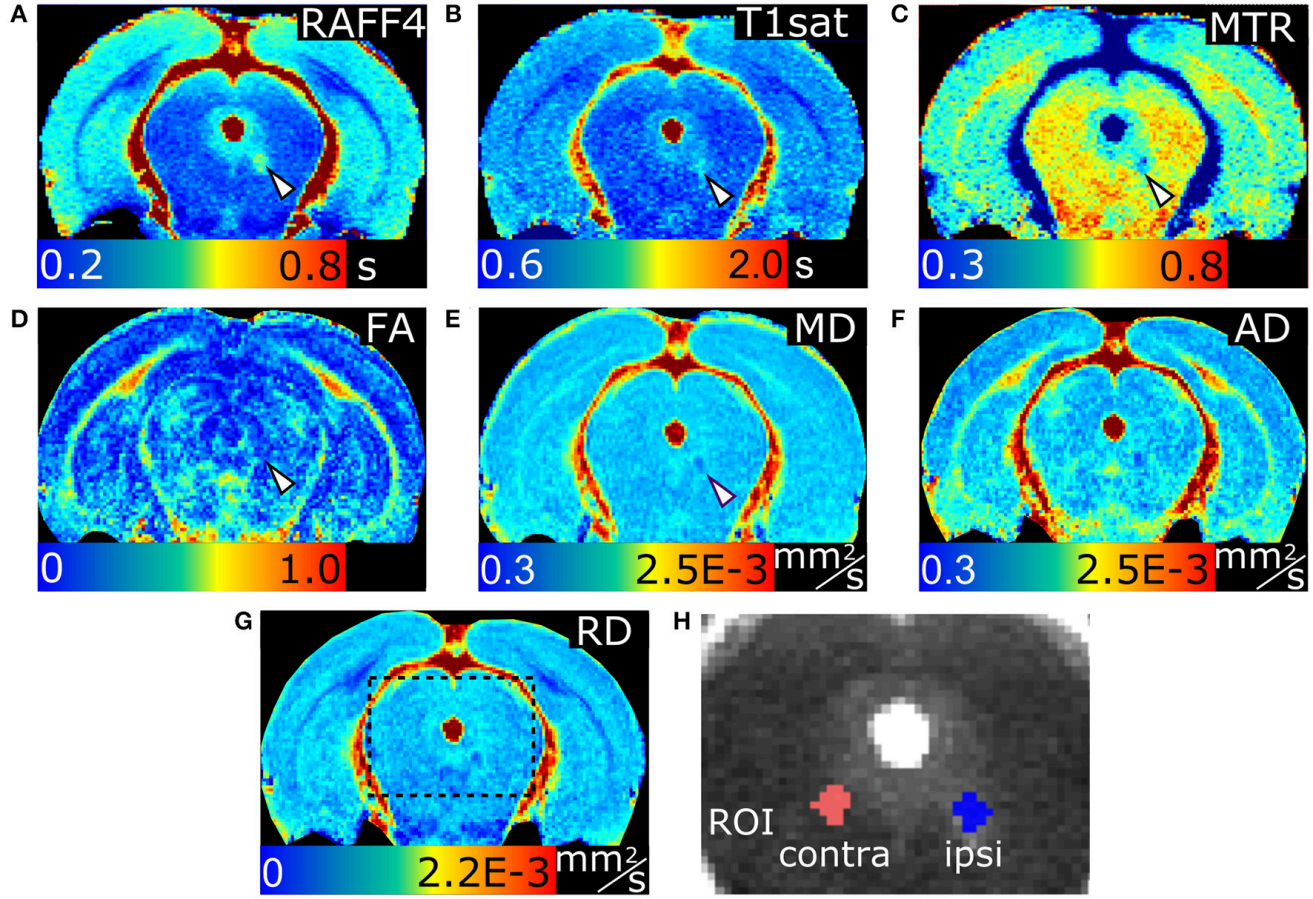
Relaxation time constants map of RAFF4 **(A)**, T_1sat_ **(B)**, magnetization transfer ratio, MTR **(C)**, fractional anisotropy, FA **(D)**, mean diffusivity, MD **(E)**, and axial and radial diffusivity, AD and RD **(F,G)** of LPC-induced lesion in the dorsal tegmental tract. Representative examples of ROIs for analyzing lesions in the dorsal tegmental tract are shown on a grayscale RAFF4 map in **(H)**. Arrowheads indicate the lesion site.

**Figure 6:**
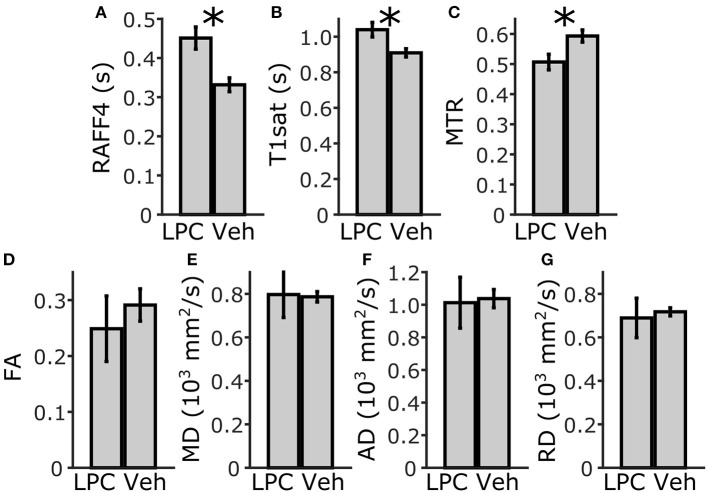
ROI analysis of MRI parameters in the dorsal tegmental tract, ipsilateral side. Mean and SD of RAFF4 **(A)**, T_1sat_ **(B)**, magnetization transfer ratio, MTR **(C)**, fractional anisotropy, FA **(D)**, mean diffusivity, MD **(E)**, and axial and radial diffusivity, AD and RD **(F,G)** obtained from LPC induced lesion ROI in the dorsal tegmental tract of LPC injected (*n* = 6) rats and from the corresponding injection site ROI in vehicle injected (*n* = 4) rats. Statistical significance; ^*^ < 0.05 (FDR adj. for testing 7 MR parameters), Mann-Whitney *U*-Test.

Lesions with a width of 0.59 ± 0.18 mm were clearly visible in RAFF4, T_1sat_ and MTR maps in the dtg (Figure 5). In ROI analysis of the dtg, RAFF4 and the MT MRI metrics showed a statistically significant difference (*p* < 0.05) between lesions induced by LPC and the corresponding area in vehicle injected animals, while the diffusion metrics did not show a significant difference (Figure 6). The RC of RAFF4 was 26.2% while the RCs of MTR and T_1sat_ were lower at 16.4 and 12.5%, respectively. Corresponding RCNRs were 6.8, 4.8, and 5.6, respectively.

#### Histology

**Figure 7:**
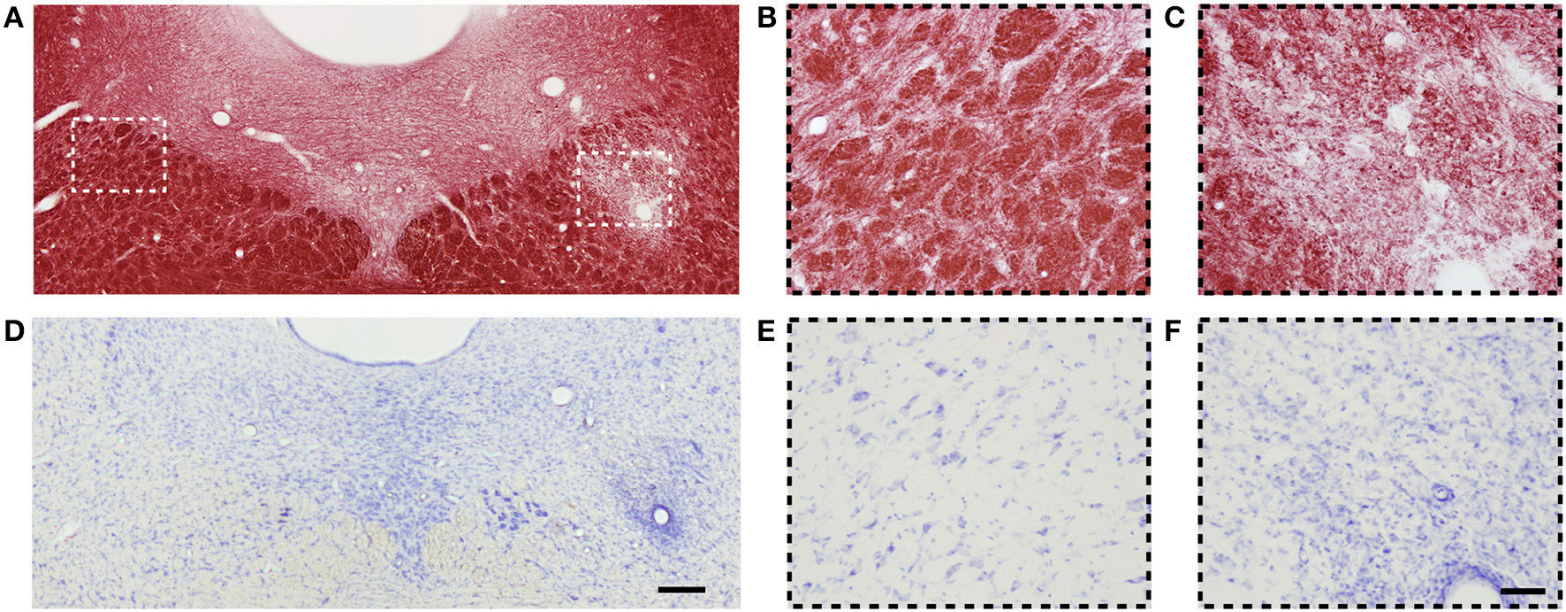
**(A)** Representative photomicrograph of myelin-stained sections of the dorsal tegmental tract. **(B,C)** are high magnification images of contra- and ipsilateral ROIs from **(A)**, respectively. **(D)** Representative photomicrographs of Nissl-stained sections of the dorsal tegmental tract. **(E,F)** are high magnification images of contra- and ipsilateral ROIs from **(D)**, respectively. Scale bars: **(D,F)** 200 and 50 μm, respectively.

Figure 7 shows myelin- and Nissl-stained sections of a rat 3 days after LPC injection in the dtg. We observed a demyelinated area ipsilaterally to the injection (Figure 7C) while the myelin content on the contralateral side appeared normal (Figure 7B).

On Nissl-stained sections, we observed an increased cell density attributed to gliosis, which overlapped with the demyelinating area (Figure 7D). On the contralateral side to the injection site, the amount and distribution of cells were normal (Figure 7E). Similarly as in the cc lesion, gliosis was diffusely distributed, and a high number of cells were found around blood vessels (Figure 7F).

### Correlation between MRI parameters and histology

**Figure 8:**
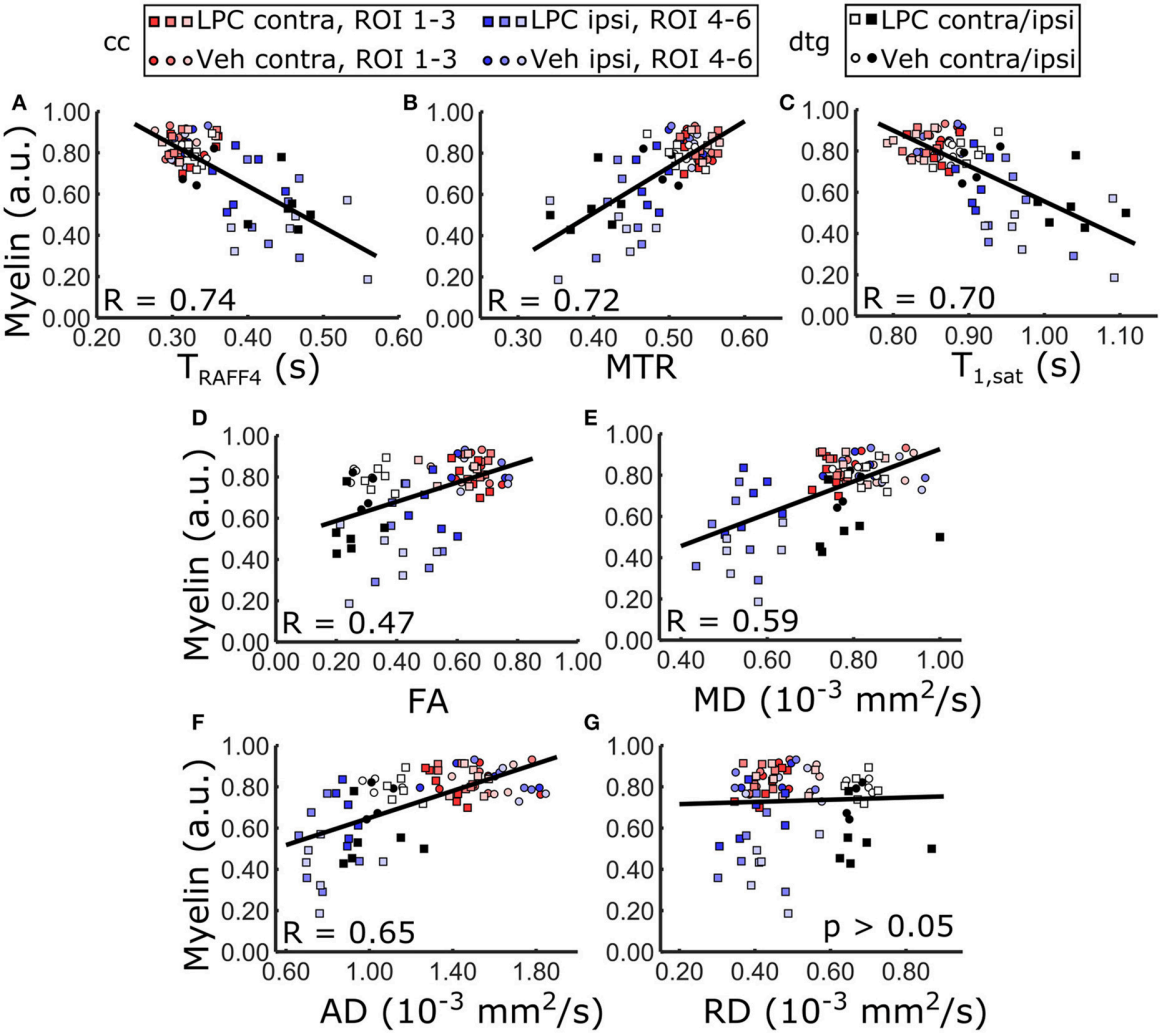
Pearson correlation between MRI metrics and optical density measured from myelin stained sections including all ROIs, i.e. LPC and vehicle injected animals, contralateral and ipsilateral ROIs. Linear regression line between optical density of myelin and **(A)** RAFF4, **(B)** MTR, **(C)** T_1sat_, **(D)** FA, **(E)** MD, **(F)** AD overlaid on the corresponding scatter plot of the ROI data. RD did not show statistically significant correlation.

RAFFn and MT parameters provided good correlation (*R* > 0.70) with myelin staining optical density when data from both brain areas and all ROIs were pooled (Table 1, Figure 8), while the correlation with myelin density was clearly lower for FA (*R* = 0.47) and for MD (*R* = 0.59). When only ROIs in the cc were included in the correlation analysis, all MRI parameters, except RD, markedly correlated (*R* > 0.66) with myelin density. However, the correlation with diffusion parameters was lower than that of RAFFn or MT despite larger relative changes in diffusion parameters than RAFFn and MT (Table 1). Importantly, in the dtg, RAFF4 and MT parameters had excellent correlation with myelin staining (*R* > 0.70), while there was no statistically significant correlation between diffusion parameters and myelin density.

**Table 1:** Pearson correlation of MRI parameters with optical density in myelin and Nissl stained sections including ipsilateral and contralateral ROIs and both LPC and vehicle injected animals.

		**RAFF4**	**MTR**	**T_1sat_**	**FA**	**MD**	**AD**	**RD**
**MYELIN**								
cc	R	−**0.742**	**0.741**	−**0.741**	**0.662**	**0.708**	**0.714**	**0.257**
	95%, lower	−0.838	0.600	−0.837	0.490	0.553	0.562	0.003
	95%, upper	−0.601	0.837	−0.599	0.784	0.815	0.820	0.480
	|upper-lower|	0.237	0.237	0.238	0.294	0.262	0.257	0.476
	p	<0.001	<0.001	<0.001	<0.001	<0.001	<0.001	0.048
dtg	R	−**0.745**	**0.719**	−**0.705**	0.438	0.122	0.279	−0.079
	95%, lower	−0.893	0.405	−0.875	–	−	−	–
	95%, upper	−0.451	0.881	−0.382	–	–	–	–
	|upper-lower|	0.442	0.476	0.493	–	–	–	–
	p	0.001	0.001	0.001	0.053	0.610	0.233	0.739
Both	R	−**0.743**	**0.719**	−**0.700**	**0.473**	**0.591**	**0.648**	0.039
	95%, lower	−0.827	0.593	−0.797	0.282	0.426	0.499	–
	95%, upper	−0.625	0.811	−0.568	0.627	0.717	0.760	–
	|upper-lower|	0.203	0.218	0.229	0.350	0.291	0.260	–
	p	<0.001	<0.001	<0.001	<0.001	<0.001	<0.001	0.732
**NISSL**								
cc	R	0.217	−0.306	0.286	−0.245	−0.169	−0.208	0.036
	95%, lower	–	–	–	–	–	–	–
	95%, upper	–	–	–	–	–	–	–
	|upper-lower|	–	–	–	–	–	–	–
	p	0.149	0.107	0.107	0.149	0.225	0.149	0.782
dtg	R	**0.587**	−**0.562**	**0.580**	−0.412	−0.298	−0.396	−0.119
	95%, lower	0.195	−0.804	0.184	–	–	–	–
	95%, upper	0.817	−0.159	0.814	–	–	–	–
	|upper-lower|	0.6224	0.645	0.629	–	–	–	–
	p	0.026	0.026	0.026	0.112	0.230	0.112	0.616
Both	R	**0.343**	−**0.450**	**0.466**	−**0.415**	−0.066	−**0.292**	**0.295**
	95%, lower	0.134	−0.610	0.274	−0.582	–	−0.481	0.081
	95%, upper	0.524	−0.256	0.622	−0.215	–	−0.078	0.484
	|upper-lower|	0.390	0.354	0.348	0.367	–	0.404	0.403
	p	0.004	<0.001	<0.001	<0.001	0.564	0.009	0.008

*Statistically significant R-values (p < 0.05, FDR adj. for testing 7 MRI parameters) are shown in bold*.

*Cc, corpus callosum; dtg, dorsal tegmental tract*.

There was a mild correlation between RAFF4 and Nissl staining intensity in the dtg (*R* < 0.59), however no statistically significant correlation in the cc and a weak correlation overall (*R* < 0.35). This indicates that cellularity is not the determining factor to contrast, but may have a small contribution for example in the case of profound gliosis (Table 1).

### Influence of pH in protein phantoms

**Figure 9:**
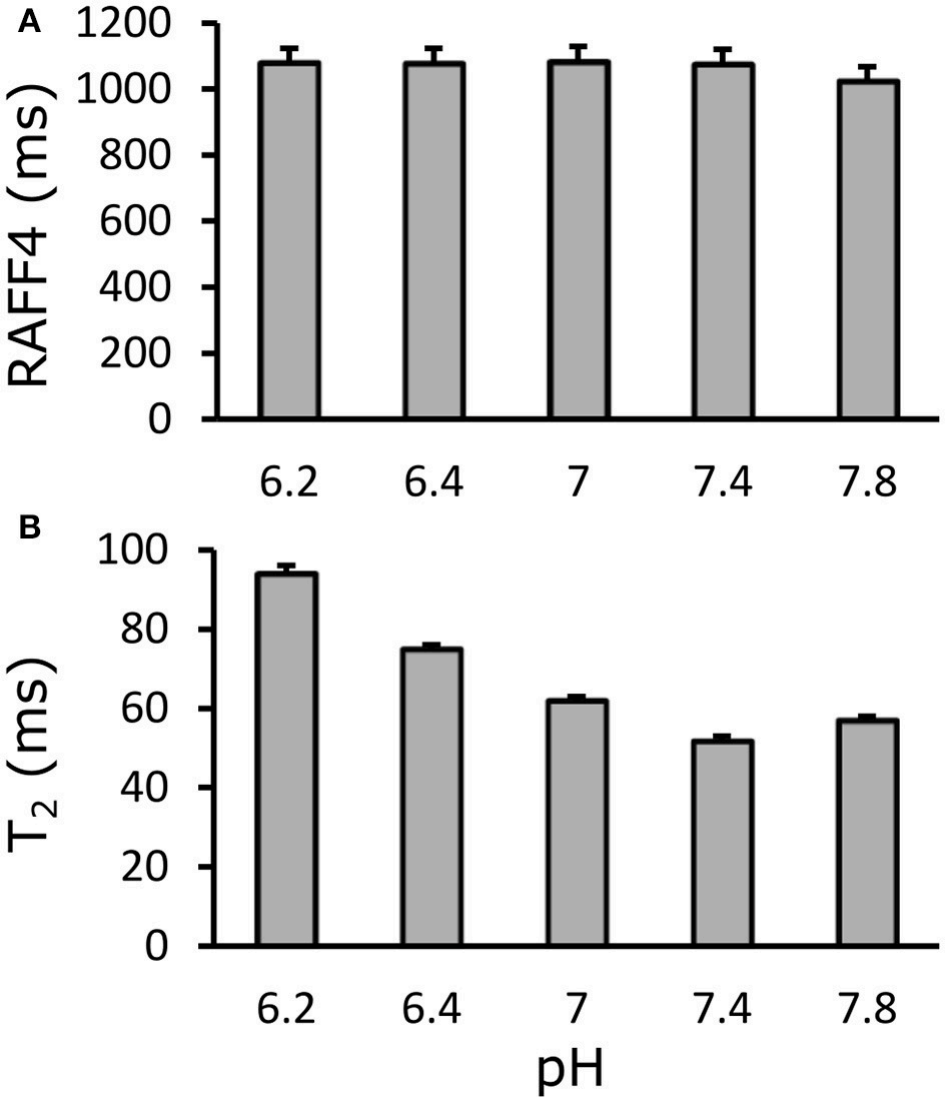
Effect of pH on **(A)** RAFF4 and **(B)** T_2_ in a bovine serum albumine phantom. RAFF4 is essentially unaffected by changing pH. Error bars indicate the standard deviation across voxels inside the ROI drawn in the phantom.

Relaxation time maps were measured with different techniques from a BSA phantom with varying pH values. While T_2_ showed clear dependence on pH, due to altered proton exchange, as expected, RAFF4 was virtually unaffected by pH at values around a physiological pH of 7.4, in the range of 6.2–7.8 (Figure 9).

## Discussion

In the present work, the value of the RAFF4 technique for detection of demyelination in the brain was investigated in a well-controlled LPC-injection rat model. Our major finding was that RAFF4 was able to robustly detect demyelination in both the cc and dtg, which have a different organization of myelinated axons and had a different amount of gliosis after LPC injection. Furthermore, RAFF4 provided excellent correlation with myelin content in both cases, while DTI was unable to reliably assess demyelinated area in the dtg, likely due to its sensitivity to underlying cytoarchitecture and gliosis. Interestingly, MT parameters measured with a recently introduced modified inversion technique (Mangia et al., 2011) were able to detect demyelination in both the cc and dtg and showed good correlation with myelin content, though the overall ability of MT at detecting demyelination was slightly lower than that of RAFF4.

Excellent correlation of RAFF4 to myelin content can be attributed to sensitivity of RAFF:n to correlation time regime in the ms-range (Satzer et al., 2015; Hakkarainen et al., 2016), which likely corresponds to exchange and dipolar interaction of myelin water and dipolar interaction with carbon-bound methylene protons. In our previous study, there was no significant difference between RAFF4 and RAFF5 in their ability to detect myelin in normal brain (Hakkarainen et al., 2016) indicating that contribution of myelin to spectral density function is relatively flat in this correlation time range. As RAFF4 has shorter relaxation time constant than RAFF5, and therefore requires shorter magnetization preparation pulse train, RAFF4 was chosen over RAFF5 for sensitizing MRI for myelin. In our previous studies, RAFF1-3 showed sensitivity to shorter correlation time regime than RAFF4-5, with reduced gray/white matter contrast and reduced sensitivity to myelin (Liimatainen et al., 2010; Satzer et al., 2015). The different sensitivities of RAFFn techniques indicate how these techniques can be tuned to detect different motional regimes corresponding to different cellular components and pathological processes.

A conventional DTI approach was used as a comparative method, as it is one of the most commonly used advanced MRI approaches in clinical settings for assessment of white matter abnormalities. In the cc, DTI was able to detect LPC lesion even better than RAFF4 based on contrast between LPC and vehicle injected animals. However, large variability of the DTI metrics led to worse contrast-to-noise ratio between the two groups in comparison to RAFF4. In addition, the pattern of diffusion changes was unexpected: decreased MD, which was associated with decreased AD, while RD was unchanged. Demyelination is classically associated with increased RD (Song et al., 2005), as water molecules can pass axonal membranes more freely in the absence of myelin. In our study, demyelination, 3 days after LPC injection, was confirmed by quantitative analysis of myelin stained histological sections. Our results indicate substantial but not complete demyelination at this subacute time point. A previous electron microscopy study in LPC injected rats showed significant thinning of the myelin sheaths around axons, but not complete dissolution of myelin especially in the early time points (Foster et al., 1980). Our TEM data showed similar changes 3 days after LPC injection. In spite of clear demyelination, some disorganized myelin sheaths were still observed, which preserved RD close to normal. Furthermore, disorganized myelin sheaths with pockets, myelin debris, and vacuoles increased the number of diffusion restricting boundaries in the intra axonal space explaining the decreased AD, FA, and MD. These findings emphasize the complexity of using simple diffusion metrics as markers for a specific pathological feature such as demyelination. On the other hand, DTI, and especially more advanced high angular resolution diffusion-weighted imaging-type diffusion imaging approaches, clearly have potential to provide information that goes far beyond the myelin content and warrants further studies. The optimal MRI protocol to assess white matter pathology should contain both a technique with high sensitivity and specificity to myelin and capability to characterize microstructural changes.

Importantly, RAFF4 and RAFF5 have been shown to correlate with myelin density to a greater extent than MT in normal brain (Hakkarainen et al., 2016). Our results here show that using advanced MTR mapping (Mangia et al., 2011), MTR can also provide excellent correlation with myelin in LPC induced demyelination although lower than that of RAFF4. In addition to anisochronous and isochronous rotating frame relaxation pathways contributing to RAFF4 and 5, RAFF4 and 5 may share with MT cross-relaxation pathways. Therefore, these two techniques could provide complementary information for characterizing tissue integrity which could be utilized by proper modeling (Underhill et al., 2011).

A benefit of the LPC model is that it causes demyelination without significant edema formation and only mild to moderate gliosis. Therefore the LPC model allows assessment of demyelination without multiple simultaneous contributing factors. In practically all human pathological conditions, demyelination is co-localized with a number of other pathological processes including inflammation, edema and acidification. There was more gliosis in the dtg than in cc after LPC injections, which likely explains why there was statistically significant correlation with Nissl staining in dtg but not in cc. However, in both cases correlation with myelin content remained good, and in general RAFF4 correlation with myelin density was higher than with other MRI methods studied. Importantly, the correlation of RAFF4 with Nissl stain proves that mechanisms other than myelin must contribute to the RAFF4 contrast. Therefore, RAFF4 is not entirely specific to myelin, as expected for any relaxation parameters that unavoidably depends on multiple biological processes. Yet, the insensitivity of RAFF4 to at least some processes such as pH changes, along with the distinct feature of RAFF4 to detect slow and ultra-slow motional regimes typical in myelin, overall suggest also a better specificity of RAFF4 to myelin as compared to other methods.

A unique feature of RAFF4 is that sensitivity to slow molecular motion can be achieved within the SAR limits of human studies, making the RAFF4 technique readily applicable to clinical settings. Indeed, excellent gray/white matter contrast with high rank RAFF4 has already been demonstrated in human brain with reasonable acquisition times (~10 min) (Liimatainen et al., 2015). While sensitivity to ms-time range can be also achieved with conventional spin-lock techniques, such as continues-wave T_1ρ_ with spin-lock field in kHz range (Sepponen et al., 1985; Gröhn et al., 2000), human studies are compromised because of high SAR of on-resonance spin-lock pulses.

## Conclusion

The excellent ability of RAFF4 to detect myelin content in healthy and pathological tissues and its insensitivity to fiber orientation distribution, gliosis and pH together with low SAR, promises RAFFn to become a useful technique for visualizing demyelinating lesions. Future work will aim to investigate later time points after LPC-induced demyelination in order to follow the remyelination of the LPC injected animals and to test specificity of RAFF4 for myelin in other disease models with more complex pathology which for instance include edema.

## Author contributions

LL, AA, and AS participated in design of the work, acquisition, analysis, interpretation of data and preparing the manuscript. LT participated in design of the work, acquisition and preparing the manuscript. LE participated in design of the work, analysis and preparing the manuscript. SMa participated in design of the work, interpretation of the data and preparing the manuscript. AN participated in design of the work and preparing the manuscript. SMi and OG participated in design of the work, analysis, interpretation of data and preparing the manuscript.

### Conflict of interest statement

The authors declare that the research was conducted in the absence of any commercial or financial relationships that could be construed as a potential conflict of interest.
